# Premature birth carries a higher risk of nephrotic syndrome: a cohort study

**DOI:** 10.1038/s41598-021-00164-2

**Published:** 2021-10-19

**Authors:** Chih-Chia Chen, Tsung Yu, Hsin-Hsu Chou, Yuan-Yow Chiou, Pao-Lin Kuo

**Affiliations:** 1 Institute of Clinical Medicine, College of Medicine, National Cheng Kung University, Tainan, Taiwan; 2 Division of Pediatric Nephrology, Department of Pediatrics, College of Medicine, National Cheng Kung University Hospital, National Cheng Kung University, 138 Sheng-Li Rd., Tainan, Taiwan; 3 Department of Public Health, College of Medicine, National Cheng Kung University Hospital, National Cheng Kung University, Tainan, Taiwan; 4 Department of Pediatrics, Ditmanson Medical Foundation, Chiayi Christian Hospital, Chia-Yi City, Taiwan; 5 Department of Bioinformatics and Medical Engineering, Asia University, Taichung, Taiwan; 6 Department of Obstetrics and Gynecology, College of Medicine, National Cheng Kung University Hospital, National Cheng Kung University, 138 Sheng-Li Rd., Tainan, Taiwan

**Keywords:** Nephrology, Kidney diseases, Glomerular diseases

## Abstract

The pathogenesis of nephrotic syndrome is unclear. We conducted a nationwide population-based cohort study to examine the associations between preterm births and subsequent development of NS. NS was defined as ≥ 3 records with ICD-9-CM codes for NS in hospital admission or outpatient clinic visits. To avoid secondary nephrotic syndrome or nephritis with nephrotic range proteinuria, especially IgA nephropathy, we excluded patients with associated codes. A total of 78,651 preterm infants (gestational age < 37 weeks) and 786,510 matched term infants born between 2004 and 2009 were enrolled and followed until 2016. In the unadjusted models, preterm births, maternal diabetes, and pregnancy induced hypertension were associated with subsequent NS. After adjustment, preterm births remained significantly associated with NS (*p* = 0.001). The risk of NS increased as the gestational age decreased (*p* for trend < 0.001). Among the NS population, preterm births were not associated with more complications (Hypertension: *p* = 0.19; Serious infections: *p* = 0.63, ESRD: *p* = 0.75) or a requirement for secondary immunosuppressants (*p* = 0.61). In conclusion, preterm births were associated with subsequent NS, where the risk increased as the gestational age decreased. Our study provides valuable information for future pathogenesis studies.

## Introduction

Nephrotic syndrome (NS) can affect pediatric population of any age, from infant to adolescent periods. Minimal change nephropathy is responsible for most cases of pediatric NS, followed by focal segmental glomerulosclerosis^1^. Although steroids can induce remission in more than 90% of patients with NS^2^, the pathogenesis is not fully understood. In recent years, several studies have explored the elevation of immunoglobulin (Ig)E and interleukin (IL)-4 and -13 in children with NS^3^. Additionally, regulatory T (T_reg_) cells have been demonstrated to play a role in minimal change nephropathy^4^, and the numbers of T_reg_ cells have been found to be reduced in patients with NS^5^. These data suggest that adaptive immune response dysfunction may play a role in NS pathogenesis.

Growing data have demonstrated that early life exposure may influence immunologic systems in later life. An early meta-analysis showed that maternal and paternal asthma is associated with offspring asthma^6^ and might impair neonatal T_reg_ cells^7^. Maternal rheumatoid diseases are associated with offspring immunological disorders^8,9^. Interestingly, Zhang *et al.* found that preterm births also affect the immune systems in later life^10^. Therefore, preterm births might influence the immune response, specifically the T cell immunity, and then predispose the onset of the NS. However, although some investigations revealed that among children with nephrotic syndrome, those born with low birth weight or premature births were associated with unfavorable clinical courses^11,12^, the relationship between preterm birth and NS development remains unknown. We hypothesized that preterm infants had a higher risk for subsequent NS and aimed to reveal their relationship in the current study. We used a nationwide population cohort to test the hypothesis due to the low incidence of NS. It should be noted that, many maternal immunologic diseases may be responsible for allergic diseases later in life^7–9^. To address this problem, we used the cohort that provided the parent–offspring linkage, which provided information on the possible impact of maternal disorders.

## Results

### Validation of NS

To decrease the possibility of misclassifications, we first validate our definition of NS. During 2004–2016, 31 patients were born between 2004 and 2009 had ≥ 3 records with ICD-9-CM codes for NS in hospital admission or outpatient clinic visits and did not have ICD-9-CM codes for secondary nephrotic syndrome or nephritis with nephrotic range proteinuria. Among them, one had IgA nephropathy, another had IgM nephropathy, and the other 29 patients had a diagnosis of NS. The positive predictive value was 93.5%.

### Demographics of study cohort

In total, 78,651 preterm infants and 786,510 matched term infants were analyzed in the current study (Table 1). The proportion of males was 57.2%. The percentage of the infants born in 2004 was 18.1%, whereas the percentage born in 2009 was 15.8%. Most preterm infants ranged in gestational age from 32 to 36 weeks (92.4%), followed by those with gestational age 29–31 weeks (4.8%). Only 2.7% were born at gestational age ≤ 28 weeks. Term infants had a higher incidence of being small for gestational age (9.8% and 6.6% in the term and preterm cohorts, respectively). Preterm infants resided in less urbanized areas and had lower family incomes and more maternal complications than term infants (both *p* < 0.001). Although the maternal and paternal ages were statistically higher in the preterm cohorts, the differences in the point estimation were low and without clinical significance.

**Table 1:** Demographic data for the preterm and matched term infants.

	Preterm (n = 78,651)	Term (n = 786,510)	*p* value
Male, n (%)	44,960 (57.2)	449,600 (57.2)	1.000
Birth year, n (%)			1.000^a^
2004	14,232 (18.1)	142,320 (18.1)	
2005	13,066 (16.6)	130,660 (16.6)	
2006	12,805 (16.3)	128,050 (16.3)	
2007	12,896 (16.4)	128,960 (16.4)	
2008	13,195 (16.8)	131,950 (16.8)	
2009	12,457 (15.8)	124,570 (15.8)	
Gestational age groups, n (%)			
≤ 28 wks	2158 (2.7)	0 (0.0)	
29–31 wks	3804 (4.8)	0 (0.0)	
32–36 wks	72,689 (92.4)	0 (0.0)	
≥ 37 wks	0 (0.0)	786,510 (100.0)	
SGA, n (%)	5202 (6.6)	76,769 (9.8)	< 0.001***
Urbanization level, n (%)			< 0.001***^b^
1 or 2 (lowest)	7374 (9.4)	63,834 (8.1)	
3 or 4	13,574 (17.3)	130,050 (16.5)	
5	18,705 (23.8)	182,671 (23.2)	
6	23,305 (29.6)	241,865 (30.8)	
7	15,693 (20.0)	168,090 (21.4)	
Family income, quartile, n (%)			< 0.001***^c^
Dependent or first	22,698 (28.9)	185,418 (23.6)	
Second	20,127 (25.6)	202,274 (25.7)	
Third	18,422 (23.4)	198,856 (25.3)	
Fourth	17,404 (22.1)	199,962 (25.4)	
Maternal age, year, mean (SD)	29.8 (5.2)	29.4 (4.8)	< 0.001***
Paternal age, year, mean (SD)	33.0 (5.7)	32.6 (5.3)	< 0.001***
Maternal complication, n (%)			
RA	139 (0.2)	994 (0.1)	< 0.001***
SLE	427 (0.5)	1852 (0.2)	< 0.001***
DM	1819 (2.3)	7246 (0.9)	< 0.001***
GDM	8855 (11.3)	81,927 (10.4)	< 0.001***
Chronic HTN	2600 (3.3)	6134 (0.8)	< 0.001***
PIH	5266 (6.7)	14,631 (1.9)	< 0.001***
Asthma	401 (0.5)	2635 (0.3)	< 0.001***

The family income was assessed at birth and was classified into four quartiles based on each birth year. PIH was defined as hypertension that complicated pregnancy after 20 weeks of gestation.

*DM* diabetes mellitus, *GDM* gestational diabetes mellitus, *HTN* hypertension, *PIH* pregnancy-induced hypertension, *RA* rheumatoid arthritis, *SGA* small for gestational age, *SLE* systemic lupus erythematosus.

^a^The *p* value implied the differences of the birth year distributions between preterm and term infants.

^b^The *p* value implied the differences of urbanization distributions between preterm and term infants.

^c^The *p* value implied the differences of family income distributions between preterm and term infants.

***p < 0.001.

### Comparison of the incidence of subsequent nephrotic syndrome in the different gestational age groups

Overall, 50 children in the preterm cohort and 285 children in the term cohort developed NS, with incidence rate of 7.1 and 4.0 per 100,000 person-years, respectively (Table 2). Preterm infants had a higher incidence of NS than term infants, and the crude hazard ratio (HR) was 1.76 (95% CI 1.30–2.38). In the univariable analyses, the NS incidence increased with decreased gestational age (HR 1.48, 95% CI 1.06–2.07; HR 4.40, 95% CI 1.96–9.87; and HR 6.41, 95% CI 2.88–16.89 for gestational ages 32–36 weeks, 29–31 weeks and ≤ 28 weeks, respectively; *p* for trend < 0.001). Interestingly, in the univariable but not multivariable models, maternal diabetes was found to potentially predispose offspring to NS (HR 2.33, 95% CI 1.15–4.69), and the offspring of mothers with pregnancy-induced hypertension also had a higher NS incidence (HR 1.75, 95% CI 1.01–3.05). After adjusting for the possible risk of NS, the preterm births remained significantly associated with subsequent NS development (adjusted HR [aHR] 1.70, 95% CI 1.24–2.34). Similarly, this condition was even more evident in the lower gestational age group (aHR 1.48, 95% CI 1.04–2.10; aHR 3.85, 95% CI 1.62–9.13; and aHR 6.16, 95% CI 2.28–16.65 for gestational ages 32–36 weeks, 29–31 weeks and ≤ 28 weeks, respectively and *p* for trend < 0.001). When we set the gestational age as the continuous variable, the hazard ratio also consistently decreased with increases in gestational age (HR 0.90, 95% CI 0.85–0.96; aHR 0.90, 95% CI 0.85–0.97).

**Table 2:** Comparison of the incidence of subsequent nephrotic syndrome in the different gestational age groups.

	NS numbers	Person-years	Incidence (per 100,000 person-years)	Crude HR	*p* value	Adjusted HR^#^	*p* value
Preterm							
Yes	50	707,104	7.1	1.76 (1.30, 2.38)***	< 0.001***	1.70 (1.24, 2.34)**	0.001**
No	285	7,097,877	4.0	Ref		Ref	
Gestational age							
≤ 28 wks	5	18,333	27.3	6.41 (2.88, 16.89)***	< 0.001***	6.16 (2.28, 16.65)***	< 0.001***
29–31 wks	6	33,778	17.8	4.40 (1.96, 9.87)***	< 0.001***	3.85 (1.62, 9.13)**	0.002**
32–36 wks	39	654,993	6.0	1.48 (1.06, 2.07)*	0.021*	1.48 (1.04, 2.10)*	0.031*
≥ 37 wks	285	7,097,877	4.0	Ref		Ref	
* p* for trend^##^					< 0.001***		< 0.001***
Gestational age				0.90 (0.85 ~ 0.96)**	0.001**	0.90 (0.85 ~ 0.97)**	0.003**
Maternal diabetes							
Yes	8	81,044	9.9	2.33 (1.15, 4.69)*	0.018*	1.69 (0.76 ~ 3.77)	0.20
No	327	7,723,937	4.2	Ref		Ref	
Maternal PIH							
Yes	13	176,625	7.4	1.75 (1.01, 3.05)*	0.048*	1.53 (0.87 ~ 2.73)	0.14
No	322	7,628,356	4.2	Ref		Ref	

PIH was defined as hypertension that complicated pregnancy after 20 weeks of gestation.

^#^Adjusted for SGA, urbanization, family income, paternal age, maternal age and maternal complications. ^##^ p for trend was to test the effect of varying gestational age on the incidence of nephrotic syndrome.

*p < 0.05, **p < 0.01, ***p < 0.001.

**Figure 1:**
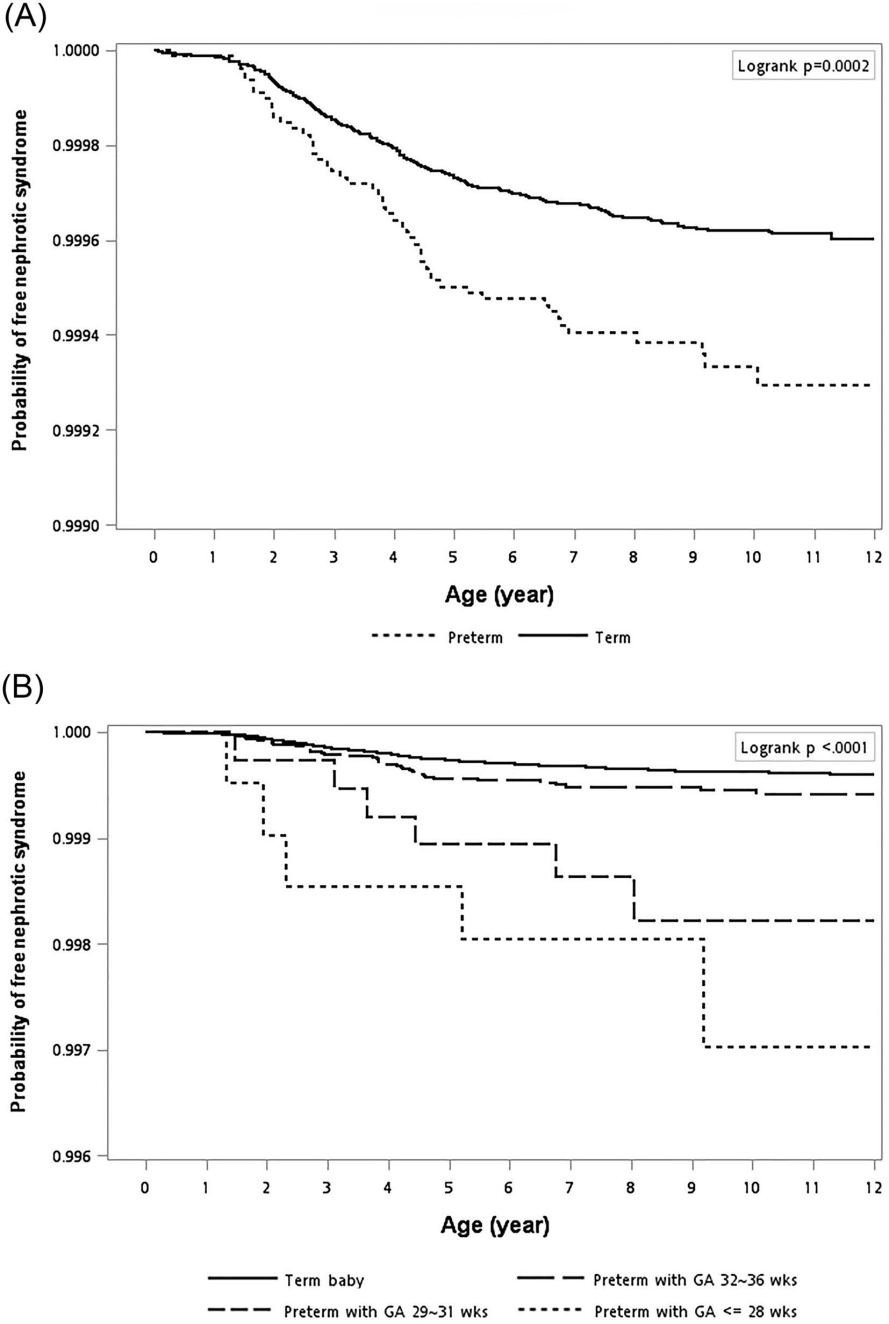
Probability of nephrotic syndrome free survival. The Kaplan–Meier nephrotic syndrome-free survival curves were according to (**A**) preterm and term infants and (**B**) the different gestational age groups. Due to data confidentiality, the number of individuals followed up for each time interval cannot be shown.

**Figure 2:**
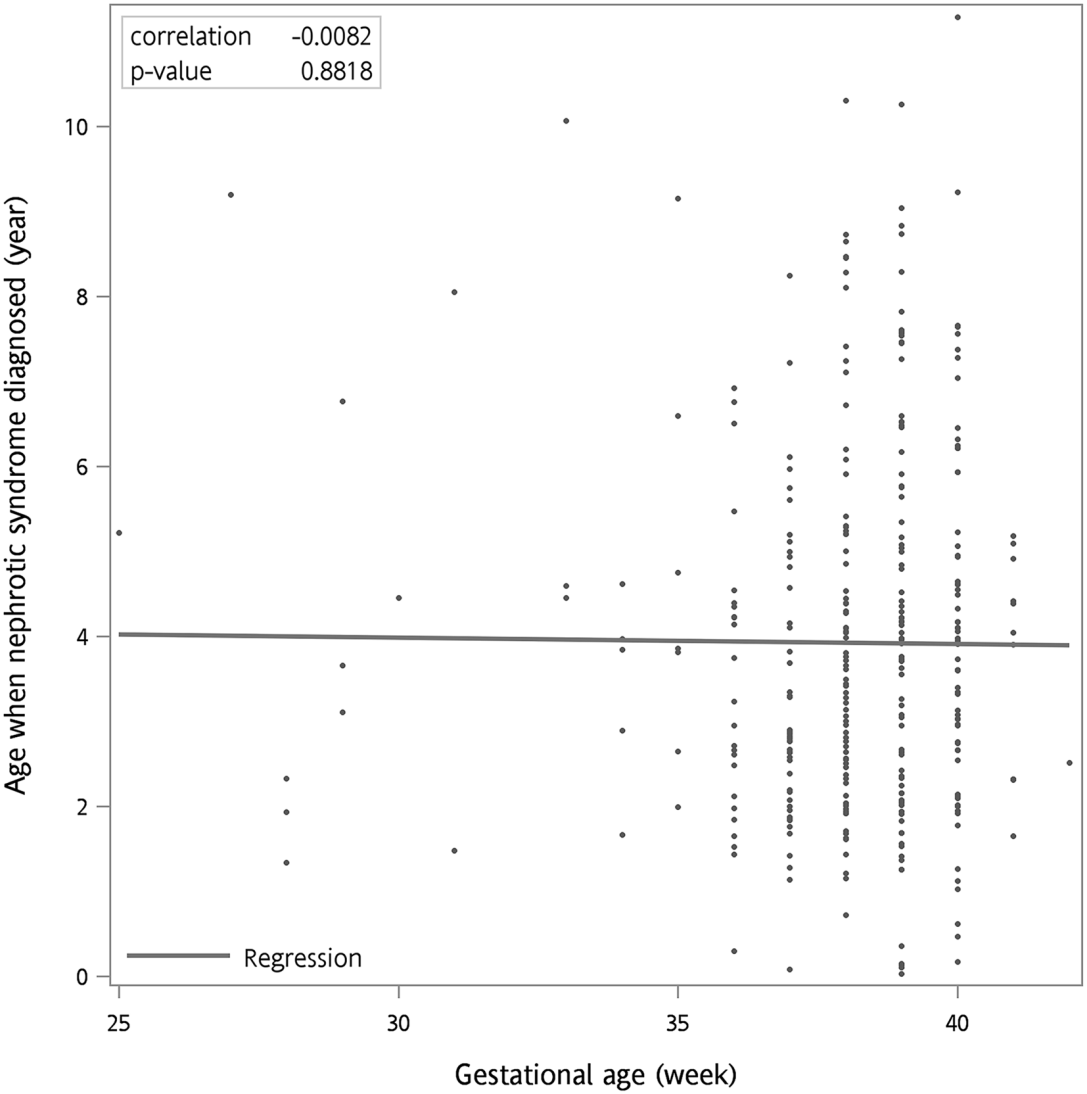
Relationships between gestational age and the age when nephrotic syndrome diagnosed. The Pearson correlation analyses showed that the correlation coefficient between gestational age and the age when nephrotic syndrome diagnosed was low.

Figure 1 is a Kaplan–Meier plot used to show the NS-free survival probability. The curve demonstrated that the preterm births were associated with a higher risk of subsequent development of NS (Fig. 1A, log-rank test, *p* < 0.001). In addition, if we grouped the preterm cohorts according to the different gestational ages, the condition was even more evident in the lower gestational age groups (Fig. 1B, log-rank test, *p* < 0.001). We also examined the relationship between gestational age and the age when nephrotic syndrome diagnosed. The Pearson correlation analyses showed that the correlation coefficient is low between gestational age and the age when nephrotic syndrome diagnosed (*r* = − 0.008, *p* = 0.88) (Fig. 2).

### Comparison of nephrotic syndrome complications in preterm and term infants

We further examined whether preterm NS patients had more complications, such as hypertension, serious infections, or ESRD. In the patients with NS during the study period, we found that 10 patients developed hypertension, 40 patients had serious infections and 5 patients had ESRD. The results showed that the odds for hypertension and serious infections were comparable in the preterm and term children (OR 2.54, 95% CI 0.63–10.15 and OR 1.24, 95% CI 0.48–2.84for hypertension and serious infections, respectively) (Table 3). The odds for ESRD were also similar between children with preterm or term births (OR 1.43, 95% CI 0.16–13.10). A total of 104 patients required secondary immunosuppressants other than steroids for better NS control, and it was determined that preterm births may not predispose children to secondary immunosuppressant usage (OR 0.84, 95% CI 0.43–1.64). The admission frequency was also comparable between the preterm and term cohorts (1.08 and 0.84 times per year in the preterm and term cohorts, respectively, *p* = 0.52). We further tested the correlation between gestational age and complications. The gestational ages were comparable between patients with and without complications (*p* = 0.73, 0.31, and 0.86 for hypertension, serious infections and ESRD, respectively) (Table 4). The gestational age was also similar between patients requiring secondary immunosuppressant usage or not (*p* = 0.91).

**Table 3:** Odds ratio for nephrotic syndrome complications and use of immunosuppressants other than steroids.

	Preterm	Incidence (per 100 infants)	Term (ref)	Incidence (per 100 infants)	OR	*p* value
Hypertension	3	6	7	2.5	2.54 (0.63, 10.15)	0.19
Serious infections	7	14	33	11.6	1.24 (0.48–2.84)	0.63
Secondary immunosuppressants usage	14	28	90	31.6	0.84 (0.43, 1.64)	0.61

*ESRD* End stage of renal disease.

**Table 4:** Comparison of gestational ages between nephrotic syndrome patients with and without secondary immunosuppressants usage or complications.

	Hypertension (n = 10)	No hypertension (n = 325)	*p* value
Gestational age, median (IQR)	39 (36–39)	38 (37–39)	0.73

	Serious infections (n = 40)	No Serious infections (n = 295)	
Gestational age, median (IQR)	38 (37–39)	38 (37–39)	0.31

	ESRD (n = 5)	No ESRD (n = 330)	
Gestational age, median (IQR)	39 (38–39)	38 (37–39)	0.86

	Secondary immunosuppressants usage (n = 104)	No secondary immunosuppressants usage (n = 231)	
Gestational age, median (IQR)	38 (37–39)	38 (37–39)	0.91

*ESRD* End stage of renal disease.

## Discussion

In the current investigation, we conducted a national population-based cohort study to examine the association between preterm birth and subsequent development of NS. Our results showed that even after extended adjustments for sex, gestational age, urbanization, family income, parental age, and maternal comorbidities, preterm infants are still significantly more at risk for developing NS. Furthermore, we found that the NS incidence increased as gestational age decreased, supporting a robust link between preterm births and NS development. Additionally, in the NS population, preterm births were not associated with more complications or higher requirements for nonsteroidal immunosuppressant drugs. Collectively, these results provide new information for future pathogenesis studies of NS.

Previous analyses have elucidated that in the NS population, children who are small for gestational age are associated with adverse clinical course, including higher incidence of steroid dependence, steroid resistance, and a need for antihypertensive drugs and other immunosuppressants^11,13^. The authors suggested that nephron deficits may be responsible for these conditions. Children with low birth weight have also been associated with secondary FSGS development and podocytopenia^1,14,15^. Despite this evidence from the NS population, whether preterm births contribute to NS development in the general population is still unclear. Because of the low incidence of NS, testing the NS incidence with limit-sized cohorts in preterm and term infants will result in insufficient power. Additionally, because a hospital-based cohort is not representative of the general population, establishing a control group with nephrotic syndromes is very complicated. To overcome this issue, we used the nationwide population-based TMCHD for the current study, which provides a great deal of information, including the perinatal and subsequent life-long medical records of the offspring linked to the parental data. Because the database covers 99% of the entire population in Taiwan, the cohorts are representative and make it possible to prevent bias by conducting cohort studies rather than case–control studies. Additionally, because of the high accessibility to medical services and high coverage of Taiwan’s national insurance, the national registry was with low ascertainment bias in NS diagnoses. Our data demonstrated that prematurity is independently responsible for subsequent NS even after adjusting for confounding biases, including being small for gestational age.

Preterm births interrupt the nephrogenesis process and reduce the number of nephrons during development^16^. A lower nephron endowment has been shown to be related to hypertension^17^. Preterm birth has also been shown to be a risk factor for development of chronic kidney disease later in life^18^. We wondered whether the lower nephron numbers were the only reason for the subsequent NS development. Therefore, we examined the proportion of patients with adverse clinical courses, including hypertension, serious infections, ESRD, secondary immunosuppressants requirement, and hospitalization frequencies. In contrast to previous data suggesting that low birth weight may predispose patients to requirements for antihypertensive drugs and may cause steroid-resistant nephrotic syndrome^13^, our results showed that the proportion of patients requiring nonsteroidal immunosuppressant drugs was similar in the preterm and term groups. Moreover, the complications of the two groups were also similar. The gestational age distributions between NS patients with or without complications were further examined and were similar. These results collectively suggest that some other pathogenesis exists in addition to loss of nephrons. These results can also be used as a reference for parents with preterm infants with NS.

The underlying mechanisms for the relationship between preterm birth and development of NS development are not known. Previous studies suggest that adaptive FSGS and podocytopenia may be the reason^1,14,15^. However, some investigations have revealed no significant pathologic differences in the small for gestational age and adequate for gestational age groups^13^. Our data analysis showed the same results, even after adjusting for small for gestational age. Additionally, the onset ages of adaptive FSGS may be later in life^19^, while the Kaplan–Meier survival curve and the time-event analysis in the current study showed that the preterm infants had a higher risk of NS at an early age. Some other mechanisms, such as immunologic response, may therefore play a role^20^. An early study demonstrated the association of type 2 helper (T_H_2)-associated cytokines and childhood nephrotic syndrome relapse^21^. Jacques et al*.* also elucidated that T_H_2 polarization precedes development of nephrotic syndrome in animal models^22^. Additionally, T_reg_ cells have been proven to be associated with NS^4,5^. Few studies have examined the differences in long-term T cell subset development in preterm and term infants. One recent work involved the use of an animal model. The result demonstrated a more systemic T_H_2 skewed pattern in preterm pigs than in term pigs with the same corrected age^23^.

T_reg_ cell proportions are higher in preterm infants at birth^24^. In contrast, preterm infants may be exposed to other risk factors for T cell dysfunction, including prenatal steroids, chorioamnionitis, stress, postnatal dysbiosis, and infection. Prenatal steroids can cause a reduced fetal thymus and have been shown to influence thymocyte development in both mice and humans^25^. A recent investigation also revealed that prenatal betamethasone may change the T cell receptor repertoire and may induce autoimmunity^26^. Claire et al*.* found that although preterm birth did not affect T_reg_ frequency, it may cause decreased T_reg_ functions^27^. Interestingly, the data from the ICESTORM project demonstrated that stress could elevate T_H_2 cytokine expression^28^. In addition, preterm infants have a higher risk of infections and exposure to antibiotics, and perinatal antibiotic exposure can influence the microbiota and is associated with allergic diseases^29^. Neonatal gut dysbiosis has also been explored to induce CD4^+^ T cell dysfunction^30^.

Similarly, a previous study also showed that maternal diabetes is associated with the development of asthma in offspring. These authors suggested that disruption in the development of the fetal immune system may be the reason for this relationship^31^. Likewise, a recent investigation revealed that exposure to maternal type 2 diabetes may contribute significantly to asthma later in life. This phenomenon has also been observed in exposure to gestational diabetes requiring medication but not in exposure to gestational diabetes that didn’t require medication^32^.

Some limitations of the current study should be mentioned. First, we used the ICD-9 code for the definition of NS and the birth weight from the TMCHD for the definition of small for gestational age, which may have resulted in misclassifications. To decrease the possibility of misclassifications, we first carried out a validation for our definition of NS, for which the positive predictive value was 93.5%, which was higher than many studies using health claims data for identification of chronic kidney diseases^33^. In addition, the birth weights were from BRD and were previously validated^34^. Hypertension and ESRD may have been underreported because of the short observation periods and possible issues with misclassification. Second, because no laboratory and pathologic data are available in our database, we cannot truly know in our NS patients whether preterm births were associated with greater FSGS pathology or nephron deficits. Therefore, this requires further investigation. We also cannot determine the subtypes of NS based on steroid responsiveness. We hence used the secondary immunosuppressants usage as the surrogate of frequently-relapsing, steroid-dependent, and steroid-resistant nephrotic syndrome. Finally, because our database mainly comprised Chinese participants, whether the results can be generalized to other ethnicities requires further verification.

In conclusion, we demonstrated that preterm infants have an increased risk of NS and that its occurrence is aggravated by maternal DM. However, preterm birth may not be associated with complicated clinical NS courses. The underlying mechanisms require further investigation to elucidate their causal relationship.

## Methods

### Data sources

We used the Taiwan Maternal and Child Health Database (TMCHD) for the current investigation. The TMCHD comprises the medical claims of four Taiwanese nationwide population-based databases: the Taiwan Birth Registration Database (BRD, 2004–2014), Birth Certificate Application (BCA, 2004–2014), National Register of Death (NRD, 2004–2014), and the National Health Insurance Research Database (NHIRD, 1998–2018) and therefore provided the parent–offspring linkage^35^. All births, including both live births and stillbirths, have to be registered legally within ten days in Taiwan. Thus, the BRD provides birth information, including sex, birth weight, gestational age, and gestational numbers. It also included the parents' characteristics, such as age at delivery. The BCA contains prenatal care details and the information recorded at birth, such as Apgar scores and congenital abnormalities. The National Health Insurance (NHI) program was implemented in 1995 and provides coverage for more than 99% of the residents of Taiwan^36^. The database described above, therefore, covered 99% of the entire population in Taiwan, where very few patients were lost to follow up^37^. Most components of the TMCHD were validated^34,37^. The TMCHD uses the International Classification of Diseases, 9th Revision, Clinical Modification (ICD-9-CM) as the diagnostic codes. This study was approved by the Institutional Review Board of National Cheng Kung University Hospital (A-ER-108-245) and all research was performed in accordance with the Declaration of Helsinki. Due to privacy regulations, information on the beneficiaries was anonymized before being released to the researchers; therefore, the Institutional Review Board of National Cheng Kung University Hospital approved informed consent was not required.

### Study cohort and outcome measurements

A nationwide retrospective cohort study design was conducted. We identified live preterm infants (gestational age < 37 weeks) born in Taiwan between January 1, 2004, and December 31, 2009, as the preterm cohort. Due to the low incidence of NS, ten randomly selected live term infants were matched by sex and birth year for each preterm infant as the comparison cohort^38^. The observational period was between January 1, 2004, and December 31, 2016. Our primary interest was whether prematurity was associated with NS and whether it was related to chronic diseases in the mother. To improve the diagnostic accuracy, NS was defined as ≥ 3 records with ICD-9-CM codes for NS, which were listed in Supplemental Table 1, in hospital admission or outpatient clinic visits. To avoid including secondary nephrotic syndrome or other nephritis with nephrotic range proteinuria, especially the IgA nephropathy, we excluded patients with any associated ICD-9-CM codes (Supplemental Table 1).

We also examined whether prematurity was responsible for more comorbidities in the NS population for the secondary outcomes. We defined hypertension when patients with related codes for ≥ 2 records in an inpatient or ambulatory claims diagnosis field (Supplemental Table 1). The serious infections were defined as patients with related codes ≥ 1 records in an inpatient claims diagnosis field (Supplemental Table 1). End-stage renal disease (ESRD) was defined as ICD-9 codes of 585 in the Registry for Catastrophic Illness Patients database.

Patients treated with immunosuppressant drugs other than steroids, including levamisole, cyclosporine, tacrolimus, cyclophosphamide, and chlorambucil, were identified. Ultimately, the admission frequency after the diagnosis of NS was examined as the proxy for disease severity.

### Validation of NS

To prevent misclassification bias, we conducted the validation analysis using National Cheng Kung University Hospital claims database. This hospital is a tertiary medical center in southern Taiwan. This database was set for reimbursement and therefore was similar to the NHIRD. All patients born between January 1, 2004, and December 31, 2009, who satisfied the NS criteria mentioned above from January 1, 2004, to December 31, 2016, were enrolled. Patients with ICD-9-CM codes of secondary nephrotic syndrome or other nephritis with nephrotic range proteinuria were excluded due to possible secondary nephrotic syndrome. Two pediatric nephrologists, Chih-Chia Chen and Yuan-Yow Chiou, reviewed the clinical records to verify the diagnosis of NS. The process of the validation study was also approved by the Institutional Review Board of National Cheng Kung University Hospital (A-ER-108-245).

### Covariates

The potential confounding factors were divided into three categories: perinatal, parental sociodemographic, and maternal medical factors. The perinatal factors include small for gestational age, which was defined as birthweight below the 10th percentile for gestational age^1^. The parental sociodemographic factors include maternal and paternal age at delivery, family income, and urbanization. The family income was assessed at birth and was classified into four quartiles based on each birth year and the levels of urbanization were based on the previous published methods^39^.

Finally, some maternal conditions were associated with allergic responses in offspring, such as diabetes^32^, hypertensive diseases^40^, rheumatoid arthritis^8^, asthma^6^, and systemic lupus erythematosus^9^. We therefore carried out extended adjustments for these factors to avoid any confounding bias. Pregnancy induced hypertension was defined as hypertension that complicated pregnancy after 20 weeks of gestation.

### Statistical analyses

We compared the characteristics of the preterm cohort to the comparison cohort using the t-test or Mann–Whitney U test for continuous factors and the chi-square or Fisher’s exact tests for categorical variables, according to variable distributions. We used Kaplan–Meier methods to demonstrate the NS-free survival probability and log-rank test to analyze the differences between distinct gestational age groups. The Pearson correlation analyses were used to examine the relationship between gestational age and the age when nephrotic syndrome diagnosed. The univariable Cox proportional-hazards model was used to test the association between preterm births and NS. To avoid confounding factors, we conducted a multivariable model to evaluate the relationship between NS and preterm birth and adjusted for gestational age, small for gestational age, urbanization, family income, maternal and paternal age, and maternal complications. We also evaluated whether preterm birth was associated with more complications in NS patients using logistic regression models.

The statistical analyses were performed with SAS statistical software (SAS Institute Inc), version 9.4. Statistical significance was defined as two-tailed *p* < 0.05.

## Supplementary Information

Supplementary Information.

## Data Availability

The data that support the findings of this study are available from Taiwan Maternal and Child Health Database (TMCHD) but restrictions apply to the availability of these data, which were used under license for the current study, and so are not publicly available. Data are however available from the authors upon reasonable request and with permission of TMCHD.
