# Supplementary material for: Premature birth carries a higher risk of nephrotic syndrome: a cohort study
Source: Sci Rep. 2021 Oct 19;11:20639. doi: 10.1038/s41598-021-00164-2 (PMC8526683; doi:10.1038/s41598-021-00164-2)
Supplement: Supplementary file 1 — Supplementary Information. [file 41598_2021_164_MOESM1_ESM.docx]

**Supplemental Table 1 ICD-9-CM diagnosis codes used in this article.**

**Codes for NS**

| 581.0 | Nephrotic syndrome with lesion of proliferative glomerulonephritis. |
| --- | --- |
| 581.1 | Nephrotic syndrome with lesion of membranous glomerulonephritis |
| 581.2 | Nephrotic syndrome with lesion of membranoproliferative glomerulonephritis |
| 581.3 | Nephrotic syndrome with lesion of minimal change glomerulonephritis |
| 581.9 | Nephrotic syndrome with unspecified pathological lesion in kidney |

**Codes for secondary nephrotic syndrome or other nephritis with nephrotic range proteinuria**

| 287 | Purpura and other hemorrhagic conditions |
| --- | --- |
| 580 | Acute glomerulonephritis |
| 580.0 | Acute glomerulonephritis with lesion of proliferative glomerulonephritis |
| 580.4 | Acute glomerulonephritis with lesion of rapidly progressive glomerulonephritis |
| 580.8 | Acute glomerulonephritis with other specified pathological lesion in kidney |
| 580.9 | Acute glomerulonephritis with unspecified pathological lesion in kidney |
| 583 | Nephritis and nephropathy not specified as acute or chronic |
| 583.0 | Nephritis and nephropathy, not specified as acute or chronic, with lesion of proliferative glomerulonephritis |
| 583.1 | Nephritis and nephropathy, not specified as acute or chronic, with lesion of membranous glomerulonephritis |
| 583.2 | Nephritis and nephropathy, not specified as acute or chronic, with lesion of membranoproliferative glomerulonephritis |
| 583.4 | Nephritis and nephropathy, not specified as acute or chronic, with lesion of rapidly progressive glomerulonephritis |
| 583.6 | Nephritis and nephropathy, not specified as acute or chronic, with lesion of renal cortical necrosis |
| 583.7 | Nephritis and nephropathy, not specified as acute or chronic, with lesion of renal medullary necrosis |
| 583.8 | Nephritis and nephropathy not specified as acute or chronic with other specified pathological lesion in kidney |
| 583.9 | Nephritis and nephropathy, not specified as acute or chronic, with unspecified pathological lesion in kidney |
| 710 | Diffuse diseases of connective tissue |
| 710.0 | Systemic lupus erythematosus |
| 710.1 | Systemic sclerosis |
| 710.2 | Sicca syndrome |
| 710.3 | Dermatomyositis |
| 710.4 | Polymyositis |
| 710.5 | Eosinophilia myalgia syndrome |
| 710.8 | Other specified diffuse diseases of connective tissue |
| 710.9 | Unspecified diffuse connective tissue disease |

**Codes for hypertension**

| 401 | Essential hypertension |
| --- | --- |
| 401.0 | Malignant essential hypertension |
| 401.1 | Benign essential hypertension |
| 401.9 | Unspecified essential hypertension |
| 402 | Hypertensive heart disease |
| 402.0 | Malignant hypertensive heart disease |
| 402.1 | Benign hypertensive heart disease |
| 402.9 | Unspecified hypertensive heart disease |
| 403 | Hypertensive chronic kidney disease |
| 403.0 | Malignant hypertensive renal disease |
| 403.1 | Benign hypertensive renal disease |
| 403.9 | Unspecified hypertensive renal disease |
| 404 | Hypertensive heart and chronic kidney disease |
| 404.0 | Malignant hypertensive heart and renal disease |
| 404.1 | Benign hypertensive heart and renal disease |
| 404.9 | Unspecified hypertensive heart and renal disease |
| 405 | Secondary hypertension |
| 405.0 | Malignant secondary hypertension |
| 405.1 | Benign secondary hypertension |
| 405.9 | Unspecified secondary hypertension |

**Codes for serious infections**

| Peritonitis | |
| --- | --- |
| 567 | Peritonitis and retroperitoneal infections |
| 567.0 | Peritonitis in infectious diseases classified elsewhere |
| 567.1 | Pneumococcal peritonitis |
| 567.2 | Other suppurative peritonitis |
| 567.3 | Retroperitoneal infections |
| 567.8 | Other specified peritonitis |
| 567.9 | Unspecified peritonitis |
| Sepsis or bacteremia | |
| 038 | Septicemia |
| 038.0 | Streptococcal septicemia |
| 038.1 | Staphylococcal septicemia |
| 038.2 | Pneumococcal septicemia |
| 038.3 | Septicemia due to anaerobes |
| 038.4 | Septicemia due to other gram‑negative organisms |
| 038.8 | Other specified septicemias |
| 038.9 | Unspecified septicemia |
| 995.9 | Systemic inflammatory response syndrome (SIRS) |
| Meningitis or central nervous system infections | |
| 320 | Bacterial meningitis |
| 320.0 | Hemophilus meningitis |
| 320.1 | Pneumococcal meningitis |
| 320.2 | Streptococcal meningitis |
| 320.3 | Staphylococcal meningitis |
| 320.7 | Meningitis in other bacterial diseases classified elsewhere |
| 320.8 | Meningitis due to other specified bacteria |
| 320.9 | Meningitis due to unspecified bacterium |
| 321 | Meningitis due to other organisms |
| 321.0 | Cryptococcal meningitis |
| 321.1 | Meningitis in other fungal diseases |
| 321.2 | Meningitis due to viruses not elsewhere classified |
| 321.3 | Meningitis due to trypanosomiasis |
| 321.4 | Meningitis in sarcoidosis |
| 321.8 | Meningitis due to other nonbacterial organisms classified elsewhere |
| 322 | Meningitis of unspecified cause |
| 322.0 | Nonpyogenic meningitis |
| 322.1 | Eosinophilic meningitis |
| 322.2 | Chronic meningitis |
| 322.9 | Meningitis, unspecified |
| 323 | Encephalitis myelitis and encephalomyelitis |
| 323.0 | Encephalitis, myelitis, and encephalomyelitis in viral diseases classified elsewhere |
| 323.1 | Encephalitis, myelitis, and encephalomyelitis in rickettsial diseases classified elsewhere |
| 323.2 | Encephalitis, myelitis, and encephalomyelitis in protozoal diseases classified elsewhere |
| 323.4 | Other encephalitis, myelitis, and encephalomyelitis due to infection classified elsewhere |
| 323.5 | Encephalitis, myelitis, and encephalomyelitis following immunization procedures |
| 323.6 | Postinfectious encephalitis, myelitis, and encephalomyelitis |
| 323.7 | Toxic encephalitis, myelitis, and encephalomyelitis |
| 323.8 | Other causes of encephalitis, myelitis, and encephalomyelitis |
| 323.9 | Unspecified causes of encephalitis, myelitis, and encephalomyelitis |
| 324 | Intracranial and intraspinal abscess |
| 324.0 | Intracranial abscess |
| 324.1 | Intraspinal abscess |
| 324.9 | Intracranial and intraspinal abscess of unspecified site |
| Cellulitis or soft tissue infections | |
| 681 | Cellulitis and abscess of finger and toe |
| 681.0 | Cellulitis and abscess of finger |
| 681.1 | Cellulitis and abscess of toe |
| 681.9 | Cellulitis and abscess of unspecified digit |
| 682 | Other cellulitis and abscess |
| 682.0 | Cellulitis and abscess of face |
| 682.1 | Cellulitis and abscess of neck |
| 682.2 | Cellulitis and abscess of trunk |
| 682.3 | Cellulitis and abscess of upper arm and forearm |
| 682.4 | Cellulitis and abscess of hand, except fingers and thumb |
| 682.5 | Cellulitis and abscess of buttock |
| 682.6 | Cellulitis and abscess of leg, except foot |
| 682.7 | Cellulitis and abscess of foot, except toes |
| 682.8 | Cellulitis and abscess of other specified sites |
| 682.9 | Cellulitis and abscess of unspecified sites |
